# Swarming Responsive Photonic Nanorobots for Motile-Targeting Microenvironmental Mapping and Mapping-Guided Photothermal Treatment

**DOI:** 10.1007/s40820-023-01095-5

**Published:** 2023-05-29

**Authors:** Luolin Li, Zheng Yu, Jianfeng Liu, Manyi Yang, Gongpu Shi, Ziqi Feng, Wei Luo, Huiru Ma, Jianguo Guan, Fangzhi Mou

**Affiliations:** 1 State Key Laboratory of Advanced Technology for Materials Synthesis and Processing, Wuhan University of Technology, Wuhan, 430070 People’s Republic of China; 2 School of Materials and Microelectronics, Wuhan University of Technology, Wuhan, 430070 People’s Republic of China; 3 School of Materials Science and Engineering, Wuhan University of Technology, Wuhan, 430070 People’s Republic of China; 4 School of Chemistry, Chemical Engineering and Life Science, Wuhan University of Technology, Wuhan, 430070 People’s Republic of China

**Keywords:** Micro/nanorobots, Collective behaviors, Responsive photonic crystals, On-the-fly sensing, Photothermal therapy

## Abstract

Micro/nanorobots can propel and navigate in many hard-to-reach biological environments, and thus may bring revolutionary changes to biomedical research and applications. However, current MNRs lack the capability to collectively perceive and report physicochemical changes in unknown microenvironments. Here we propose to develop swarming responsive photonic nanorobots that can map local physicochemical conditions on the fly and further guide localized photothermal treatment. The RPNRs consist of a photonic nanochain of periodically-assembled magnetic Fe_3_O_4_ nanoparticles encapsulated in a responsive hydrogel shell, and show multiple integrated functions, including energetic magnetically-driven swarming motions, bright stimuli-responsive structural colors, and photothermal conversion. Thus, they can actively navigate in complex environments utilizing their controllable swarming motions, then visualize unknown targets (e.g., tumor lesion) by collectively mapping out local abnormal physicochemical conditions (e.g., pH, temperature, or glucose concentration) via their responsive structural colors, and further guide external light irradiation to initiate localized photothermal treatment. This work facilitates the development of intelligent motile nanosensors and versatile multifunctional nanotheranostics for cancer and inflammatory diseases.

**Supplementary Information:**

The online version contains supplementary material available at 10.1007/s40820-023-01095-5.

## Highlights

- Responsive photonic nanorobots (RPNRs) simultaneously exhibit energetic magnetically-propelled swarming motions, bright stimuli-responsive structural colors, and photothermal conversion.
- The swarming RPNRs can actively navigate in complex environments and collectively map out local physicochemical conditions (e.g., pH, temperature, or glucose concentration) on the fly via their bright responsive structural colors.
- The swarming RPNRs can visualize an unknown target (e.g., tumor lesion) via motile-targeting mapping and then guide the external NIR light to initiate localized photothermal treatment.

**Supplementary Information:**

The online version contains supplementary material available at 10.1007/s40820-023-01095-5.

## Graphical abstract

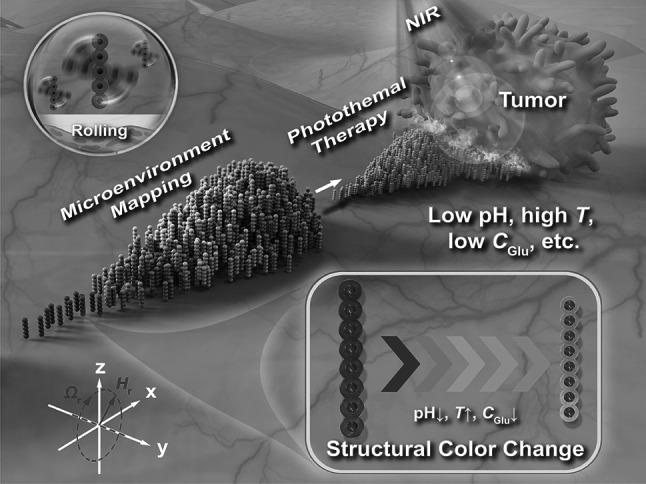

## Introduction

Physicochemical conditions of the local microenvironment, such as local viscosity, temperature, redox potential, and pH, determine the mass diffusion, energy transfer, reaction rate, and intermolecular interactions in cells, tissues, and so on [1–3]. For instance, due to abnormal metabolism in tumor cells, tumor microenvironments have been characterized by numerous physical and (bio)chemical differences relative to normal tissues, which further lead to the accelerated proliferation, migration, and invasion of tumor cells and the reduced function of immune cells [4–6]. Therefore, detecting and mapping local physicochemical conditions in biological microenvironments are of great significance for understanding various physiological processes, studying pathogenesis, and developing drugs. Up to now, various nanosensors have been developed using optical, magnetic, and electrochemical signal transduction methods [7–12]. Among them, optical nanosensors based on responsive photonic crystals, which refer to the periodic nanostructures with adjustable photonic bandgaps in response to external stimuli, have gained considerable attention recently due to their rapid visual readout, high stability, and robust reversibility [13–23]. However, traditional nanosensors generally involve no motility and can not actively explore unknown microenvironments to report local physicochemical changes.

Motile micro/nanorobots (MNRs) are capable of propelling and navigating in various liquid media by harvesting energy from surrounding chemicals or external fields [24–33]. Due to their small sizes, they hold great promise to operate in hard-to-reach cavities of the human body that are impossible by conventional techniques, and thus may bring revolutionary changes to future theranostics [27, 34, 35]. Especially, when incorporated with signal transducers, they may act as swimming micro/nanosensors to perform sensing in a motile-targeting manner, and provide new opportunities for analytical and diagnostic technologies. For example, by boosting mass/energy exchanges with surrounding environments on the fly, the MNRs have been demonstrated to efficiently detect nucleic acid, proteins, bacteria, heavy metal ions, and pH, utilizing transduction mechanisms of motion speed, fluorescence, surface-enhanced Raman scattering (SERS), and current [36–43]. However, current sensing MNRs are single MNRs relying strongly on random propulsions, and are inadequate to perform complex rapid, wide-range, and robust sensing tasks due to their low targetability, limited area coverage, and poor reproducibility (individual differences). On the other hand, purely through local diffusiophoretic, electrostatic, magnetic, and hydrodynamic interactions, MNRs can further self-organize into large swarms and show intriguing collective behaviors that single individuals do not have, such as enhanced driving forces, strong robustness, adaptive reconfigurations, rapid large-area coverage, and high imaging contrast [44–51]. Thus, the swarming MNRs can operate reliably in complex environments with excellent trackability, and are also able to cooperatively transport heavy cargoes or deliver a large dose of drugs in a short period of time [52–54]. Nonetheless, the so-far developed swarming MNR systems are composed of rigid individuals or building blocks with poor intelligence (e.g., Fe_3_O_4_ and TiO_2_ particles) [55], and usually can not perceive and report local physicochemical changes in surrounding microenvironments.

Here we propose to develop swarming responsive photonic nanorobots (RPNRs) capable of translating microenvironmental physicochemical conditions into visual color-mapping signals on the fly and further guiding localized photothermal treatment. The RPNRs consist of a nanochain of periodically-arranged Fe_3_O_4_ nanoparticles (NPs) encapsulated in a responsive hydrogel shell, and exhibit multiple integrated functions, including magnetic propulsion under alternating magnetic fields due to their magnetic response, stimuli-responsive structural colors from their periodic nanostructures and responsive hydrogel shell [18], and photothermal effect from light absorption and conversion of the Fe_3_O_4_ components [56]. With these integrated functions, the RPNRs can actively navigate in complex microenvironments utilizing their controllable collective motions, then visualize the unknown target (e.g., tumor lesion) by mapping out local abnormal physicochemical changes (e.g., pH, temperature, or glucose concentration changes) via their responsive structural colors, and further guide external near-infrared (NIR) irradiation to initiate their photothermal conversion to achieve localized photothermal treatment. This work facilitates the development of intelligent motile photonic nanosensors for targeted microenvironmental mapping, and the integrated functions of the developed swarming RPNRs may enable them to act as smart nanotheranostics for cancer and inflammatory diseases.

## Experimental Procedure

### Materials

All the chemicals used in this work were of analytical grade and were used as received without further purification. Acrylic acid (AA), 2-hydroxyethyl acrylate (HEA), 3-acrylamido phenylboronic acid (AAPBA), N-hydroxyethyl acrylamide (HEAA), N-isopropyl acrylamide (NIPAM), N-(hydroxymethyl) acrylamide (NHMA), ethylene glycol dimethacrylate (EGDMA), bis-acrylamide (BIS), 2-hydroxy-2-methylpropiophenone (HMPP), ethylene glycol (EG), dimethyl sulfoxide (DMSO), Poly(acrylic acid) (PAA) solution (50%, M.W. ~ 3,000), and ethanol were purchased from Aladdin and were used as received.

### Preparation of RPNRs

The monodisperse superparamagnetic Fe_3_O_4_ NPs with a size of 150 nm were synthesized by a modified polyol process reported previously [57]. To prepare pH-responsive photonic nanorobots (pH-RPNRs), a precursor solution was prepared at first by mixing 0.75 mg mL^−1^ Fe_3_O_4_ NPs, 300 mM AA, 127 mM HEA monomers, 13 mM crosslinker EGDMA, and 5 mM photoinitiator HMPP in 0.6 mL EG/water (5:1 in volume) solution. Then, the precursor solution was placed in a static magnetic field (*H*, 500 Gs) for 30 s. After being irradiated by UV light for 5 min and rinsed with the ethanol twice, the Fe_3_O_4_@poly(AA-co-HEA) pH-RPNRs were obtained, and then transferred into distilled water for later experiments. Using a similar procedure, temperature-responsive photonic nanorobots (T-RPNRs) comprising a photonic Fe_3_O_4_ nanochain and a poly(NIPAM-co-NHMA) hydrogel shell were prepared by using 1.15 mL PAA solution (50 mg L^−1^) precursor solution with Fe_3_O_4_ NPs (0.76 mg mL^−1^), EG (1.77 M), NIPAM (355 mM), NHMA (67 mM), BIS (4 mM), and HMPP (12 mM). Furthermore, glucose-responsive photonic nanorobots (G-RPNRs) comprising a photonic Fe_3_O_4_ nanochain and a poly(AAPBA-co-HEAA) hydrogel shell were fabricated by using 1 mL DMSO/PAA (9:1 in volume) precursor solution with Fe_3_O_4_ NPs (750 mg mL^−1^), AAPBA (25 mM), HEAA (141 mM), BIS (3 mM), and HMPP (5 mM).

### Characterization

The optical microscopy, scanning electron microscopy (SEM), and transmission electron microscopy (TEM) images were captured on an optical microscope (Leica DMI 3000M, Germany), field-emission scanning electron microscope (Hitachi S-4800, 10 kV, Japan), and high-resolution transmission electron microscope (JEOL JEM-2100F, 200 kV, Japan), respectively. The Fourier transform infrared spectroscopy (FT-IR) spectra were obtained by using a Nicolet 60-SXB FTIR spectrometer (USA). The magnetic hysteresis loop was acquired by a physical property measurement system (Model PPMS-9, Quantum Design, USA). The reflection spectra of RPNRs were captured using a fiber-optic spectrometer (Ocean Optics USB2000 + , UK). The thermal images were captured using a thermography camera (FLIR T420, USA).

### Magnetic Propulsions of RPNRs

The magnetic propulsion experiments were conducted in a customized magnetic field system consisting of electric current supplies (ATA-309 Power Amplifiers, China), a signal source (NI USB-6343, USA), and a 3-axis Helmholtz electromagnetic coil. An aqueous suspension of RPNRs at a certain concentration was added dropwise to a glass substrate or a microfluidic channel, and then a permanent magnet (3,000 Gs) was used to collect dispersed RPNRs and allow them to settle near the substrate. The RPNRs were transferred to the coil mounted on an inverted optical microscope (Leica DMI 3000M, Germany), and then activated and navigated by applying a rotating magnetic field (**H**_r_(*t*)) with different directions, strength *H*_0_, and frequency *f*. All videos were analyzed using Video Spot Tracker V08.01 and ImageJ software.

### Targeted On-the-Fly Microenvironmental Mapping

A microfluidic channel filled with 1 mL phosphate-buffered saline (PBS) buffer was used to test the targeted on-the-fly pH and glucose mapping of the swarming RPNRs. At first, RPNRs suspended in water (5 mg mL^−1^) were added dropwise (20 μL) to a pool at one end of the microchannel, and collected near the substrate using a permanent magnet. Then, test targets (agar gels with different pH values or 50 mM glucose) were placed at the other end of the microchannel. An **H**_r_(*t*) with an *H*_0_ of 25 mT and an *f* of 2 Hz was applied to activate the RPNRs to cross the microchannel, reach the test targets, and complete the on-the-fly microenvironmental mapping. The structural color changes of the RPNRs during the targeted on-the-fly mapping were recorded under dark field microscopy. The macroscopic targeted on-the-fly pH mapping was carried out in a torturous channel, and recorded by a digital camera of a smartphone. The targeted on-the-fly temperature mapping of the swarming RPNRs was conducted by driving them toward a heater (Ruibao, NR-81530, China), which was set at 45 °C and turned on after the RPNRs reached it.

### Numerical Simulation

The simulations were performed using the diffusion and laminar flow modules of COMSOL Multiphysics software. The simulation model was built up by immersing one or two pieces of agar gel in a cylindric well (radius *r* = 3 mm, height *h* = 0.5 mm) connected to a narrow canal (width *a* = 0.8 mm, *h* = 0.5 mm, and length *l* = 5 mm) (Fig. 4a, d). The well and canal were filled with water (pH = 7), and the pH at the gel surface was set to be 7.4 or 4.4. A fluidic flow with a velocity of 120 μm s^−1^ was injected from the left narrow canal into the cylindric well, simulating the flow of swarming nanorobots. The diffusion constant of H^+^ and OH^−^ was set to be 9.3 × 10^−9^ and 5.2 × 10^−9^ m^2^ s^−1^. The distribution of pH in the well and canal was obtained by simulating the diffusion of H^+^ and OH^−^ from the agar gel to the surrounding medium while being disturbed by the fluid flow.

### Photothermal Properties

2 mL aqueous suspension of pH-RPNRs with different concentrations (5.00, 2.50, 1.25, and 0 mg mL^−1^) was added to a standard cuvette and exposed to a NIR laser (808 nm, Leize BOT808-2D200F-S2, China) with different power densities of 2.0, 1.5, 1.0 and 0.5 W cm^−2^ at room temperature (25 °C) for 300 s. The temperature was recorded at 10 s intervals using a thermography camera (FLIR T420, USA).

### Cytotoxicity Study

MCF-7 cells were purchased from China Center for Type Culture Collection. The cell culture media (CCM) were prepared by using Dulbecco’s modified eagle medium (DMEM, Sigma) with a 1% penicillin/streptomycin mixture from Capricorn and 10% fetal bovine serum (FBS) from Life Technologies. All cells were incubated in an incubator at 37 °C with 5% CO_2_. All materials were UV sterilized before use. MCF-7 cells were seeded into a 96-well plate at a density of 8,000 cells per well and cultured for 24 h. Then, pH-RPNRs with different concentrations (0–2 mg mL^−1^) were added to the culture medium. After 24 h of treatment, a standard cell counting kit-8 (CCK-8) was used for the detection. The well was washed with PBS buffer three times after the removal of the culture medium. Then, each well was incubated in complete DMEM with 10% v/v CCK-8 for 2 h at 37 °C. Afterward, the absorbance of the medium was measured by a microplate reader (Multiskan GO, Thermo Scientific, USA) at 450 nm to calculate cell viability.

### Serum Stability Study

The serum solution was prepared by mixing PBS with 10% v/v FBS. Then, the serum solution was mixed with 5 mg mL^−1^ of pH-RPNRs and the mixture was placed in a 37 °C incubator. The pH-RPNRs were magnetically separated and redispersed in water after 0, 1, 2, and 4 day, respectively. The zeta potential of pH-RPNRs was measured using a NanoBrook 90Plus Zeta (USA). The dispersity of pH-RPNRs in the serum solution was observed after 1 and 2 day under a static horizontal magnetic field using the optical microscope.

### Hemolysis Assay

EDTA-anticoagulated rabbit blood was purchased from Wuhan Chundu Biotechnology Co., Ltd, China. Firstly, 2 mL of blood sample was added to 5 mL of PBS, and then red blood cells (RBCs) were isolated from serum by centrifugation at 1500 rpm for 5 min. After being washed three times with 5 mL of PBS solution, the purified blood was diluted to 1/10 of its volume with PBS solution. 0.5 mL of diluted RBC suspension was then mixed with (a) 2.5 mL of PBS as a negative control, (b) 2.5 mL of D. I. water as a positive control, and (c) 2.5 mL of pH-RPNRs suspensions at concentrations of 2.5 and 5 mg mL^−1^. Then all the mixtures were vortexed and kept at room temperature for 3 h. Finally, the absorbance of supernatants at 540 nm was determined. The percent hemolysis of RBCs was calculated as follows: hemolysis = [(sample absorbance-negative control absorbance)/(positive control absorbance–negative control absorbance)] × 100%.

### Mapping-Guided Photothermal Treatment

All materials were UV sterilized before use. At first, MCF-7 cells with a density of 60,000 cells cm^−2^ were seeded in a glass substrate with an area of about 0.1 cm^2^ for 24 h. The glass substrate was then immersed in PBS buffer with pH 6.5 and kept for 5 s. Immediately afterward, the substrate was transferred to the microchannel as a test target. Then, the pH-RPNRs (5 mg mL^−1^) were activated and navigated under an **H**_r_(*t*) (*H*_0_ = 25 mT, *f* = 2 Hz) to cross the microchannel and reach the test target. When the structural color of the pH-RPNRs turned from red to orange after covering the MCF-7 cells, a NIR light with a power density of 2.0 W cm^−2^ was applied for 300 s to trigger the photothermal conversion of the pH-RPNRs to induce cell apoptosis. The live/dead staining was used to evaluate the MCF-7 cell viability. The cells were stained with 4.5 μM propidium iodide (PI) (dead cells were labeled red) and 2 μM Calcein-AM (live cells were labeled green) for 30 min at 37 °C, and subsequently analyzed by fluorescence microscopy. For comparison, MCF-7 cells viability in the control (without pH-RPNRs and NIR irradiation), NIR-only (NIR), and pH-RPNRs-only (RPNRs) groups were also tested.

## Results and Discussion

### Design and Preparation

**Fig. 1:**
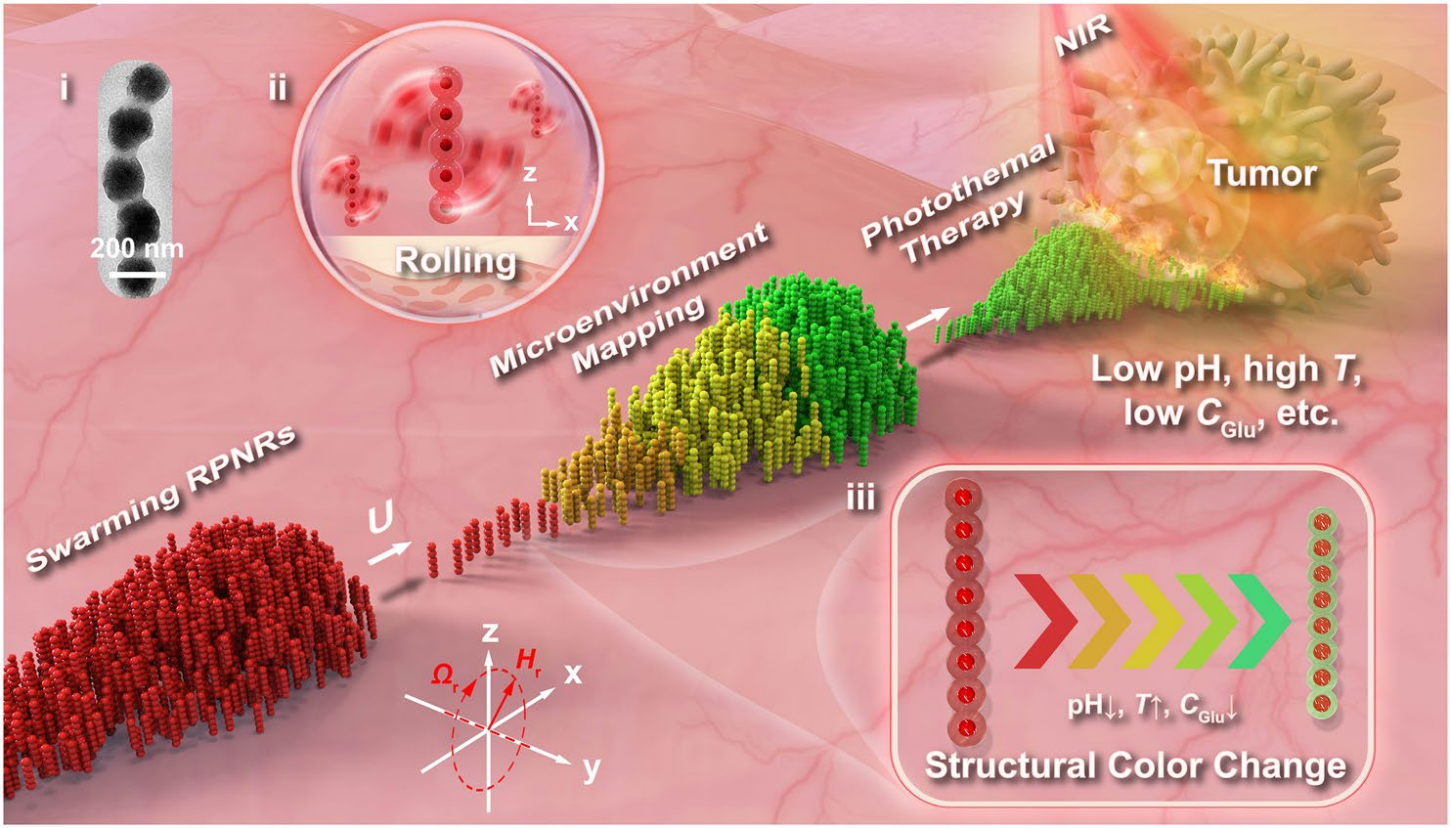
Conceptual design and functions. The responsive photonic nanorobots (RPNRs) consist of a periodic Fe_3_O_4_ nanochain encapsulated in a responsive hydrogel shell (inset i). Under a rotating magnetic field **H**_r_(*t*), they can swarm toward a target region in a rolling mode (inset ii), then collectively map local abnormal physicochemical conditions (e.g., pH, temperature, or glucose concentration) on the fly when approaching a targeted tumor site via responsive structural color changes (inset iii), and further enable mapping-guided localized photothermal tumor therapy under external near-infrared (NIR) irradiation. A superficial tumor lesion (e.g., superficial lung, oesophageal, gastric, or bladder tumor) is depicted as a typical potential application scenario

The conceptual design and functions of swarming RPNRs are shown in Fig. 1. The designed RPNR comprises a photonic nanochain of periodically-arranged magnetic Fe_3_O_4_ NPs and a responsive hydrogel shell (inset i in Fig. 1). Under a rotating magnetic field **H**_r_(*t*), the RPNRs can perform directional motions in a rolling mode due to their magnetic response (inset ii in Fig. 1), and further self-organize into microswarms utilizing local hydrodynamic interactions [47]. In addition, due to the photonic bandgap resulting from the periodic organization of the encapsulated Fe_3_O_4_ NPs and responsive swelling or deswelling of the hydrogel shell [18], the RPNRs would display bright structural colors in response to local physicochemical conditions (e.g., low pH, high temperature and low glucose concentration near tumor lesion [2]) (inset iii in Fig. 1). Utilizing their swarming motions and responsive structural colors, they can actively navigate in complex microenvironments, and visualize unknown tumor targets by mapping local abnormal physicochemical conditions in real-time and with high robustness. Further, by employing the mapping result for guidance in nanorobot-user interaction, they, due to the photothermal conversion of the Fe_3_O_4_ components under NIR light [56], can be then triggered to perform the localized photothermal treatment to induce tumor-cell apoptosis. In contrast to the reported swarming magnetic MNRs, which are mainly comprised of magnetic particles with poor intelligence and simple functions [24, 46], the designed swarming RPNRs can perceive and respond to surrounding physicochemical signals, and show multiple integrated functions of magnetically-driven collective motions, responsive structural colors, and photothermal conversion. These features bestow the swarming RPNRs with great potential in motile-targeting disease diagnosis and imaging-guided treatment. Given the considerable challenges associated with localizing and navigating nanorobots within the bloodstream, the swarming RPNRs are primarily designed for potential applications in the diagnosis and treatment of superficial tumors, such as superficial oral, esophageal, lung, gastric, and bladder carcinomas [58–60].

To implement the concept, the pH-RPNRs were fabricated at first using an in-situ magnetic assembly-polymerization method (see details in Experimental Procedure and Fig. S1a) [18]. The fabricated pH-RPNR has a nanochain-like structure with a length of ~ 10 μm and a diameter of 200 nm (Fig. S1b-d). The detailed structure of the nanorobot was confirmed by TEM observation and FT-IR analysis, suggesting that it comprises a poly(acrylic acid-co-2-Hydroxyethyl acrylate) (poly(AA-co-HEA)) hydrogel shell (8 nm) enveloping an inner one-dimensional nanochain of periodically-arranged Fe_3_O_4_ NPs (150 nm) (Fig. S1e, f). The hysteresis loop of pH-RPNRs reveals that they are superparamagnetic and have a high saturated mass magnetization of 27.4 emu g^−1^ at room temperature (Fig. S1g), facilitating their magnetically-driven propelling and swarming.

### Magnetic Propulsions and pH Responses

Under a rotating magnetic field (**H**_r_(*t*), Eq. 1) [61], a magnetic torque is exerted on the magnetic pH-RPNR and forces it to rotate around its short axis with an angular velocity *ω*_r_.

1 $$ {\mathbf{H}}_{{\text{r}}} \left( t \right) = \left( {H_{0} \sin 2\pi ft{\mathbf{n}}_{x} ,0, H_{0} \cos 2\pi ft{\mathbf{n}}_{z} } \right) $$

**Fig. 2:**
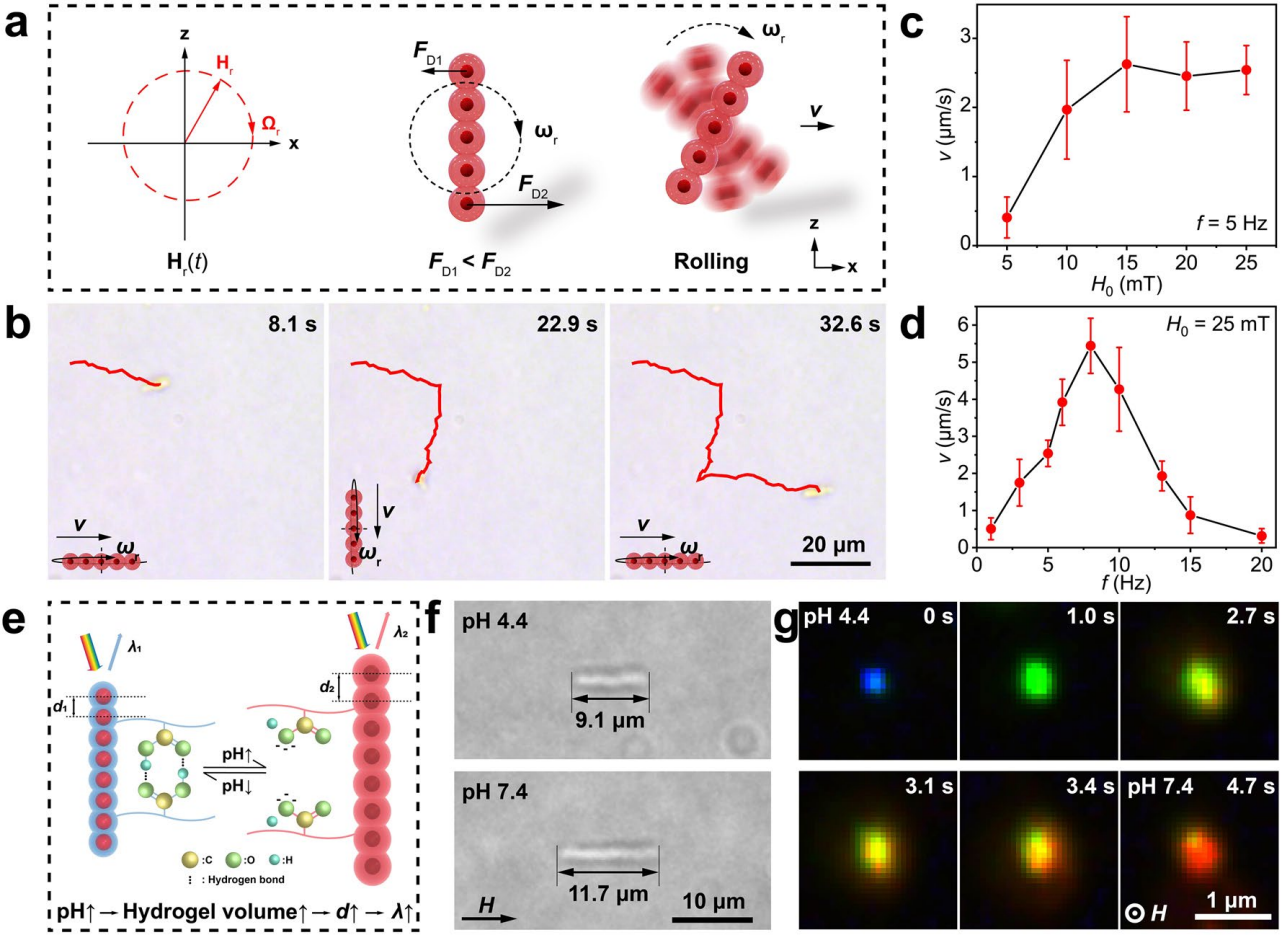
Magnetic propulsions and pH responses of single pH-RPNRs**. a** Schematic illustration of a pH-RPNR moving in a “rolling” mode under **H**_r_(*t*) with an angular frequency ${\Omega }_{\mathrm{r}}$ (${\Omega }_{\mathrm{r}}=2\pi f$). **b** Time-lapse microscopic images depicting a “rolling” pH-RPNR moving in a predesigned trajectory (red curves) when navigated by the **H**_r_(*t*). The velocity (*v*) of the rolling pH-RPNRs as a function of **c** the strength (*H*_0_) and **d** frequency (*f*), respectively. **e** Schematic illustration of the pH-induced deformation and structural color change of the pH-RPNR. **f** Optical microscopic images showing the length of a typical pH-RPNR at pH 4.4 and 7.4, respectively. **g** Time-lapse dark-field optical microscopic images depicting the structural color change of a pH-RPNR with the increasing pH under a static vertical magnetic field (*H*)

Here, *H*_0_ and $f$ are the strength and frequency of the rotating **H**_r_(*t*), ${{\varvec{n}}}_{x}$, ${{\varvec{n}}}_{y}$, ${{\varvec{n}}}_{z}$ are the unit vectors in the *x*-, *y*-, and *z*-axis directions, respectively. When rotating near a substrate (the hydrodynamic no-slip boundary), the top and bottom of the rotating pH-RPNR experience different viscous fluidic drag forces (*F*_D1_ and *F*_D2_, *F*_D1_ < *F*_D2_, Fig. 2a). As a result, the hydrodynamic symmetry of the rotating pH-RPNR is broken, and they can thus perform a “rolling” translational motion due to the coupling of the rotational motion around the bottom and the translational motion of the bottom. The detailed mechanism of the nanorobots is shown in Fig. 2a, and it depicts that a pH-RPNR rotating about the *y*-axis with the rotating **H**_r_(*t*) would move in the *x*-direction near the substrate.

The translational motion of a typical chain-like pH-RPNR is shown in Fig. 2b and Video S1. It can be seen that the pH-RPNR moves in a nearly straight-line trajectory with a *v* of 2.8 μm s^−1^ under a rotating **H**_r_(*t*) with an *H*_0_ of 25 mT and an *f* of 3 Hz (0–8.1 s in Fig. 2b). By simply changing the direction (or rotating axis) of the rotating **H**_r_(*t*), the motion direction of the pH-RPNRs can be adjusted, and it can move along a predesigned trajectory, as verified by the red zigzag trajectory with two sharp turns (8.1–32.6 s in Fig. 2b). Besides the controllable directions and trajectories, the velocity (*v*) of the pH-RPNRs can also be controlled by adjusting *H*_0_ and *f* of the applied **H**_r_(*t*). When the* H*_0_ was increased from 5 to 15 mT while keeping *f* at 5 Hz, the *v* of the pH-RPNRs increased gradually (Fig. 2c). This is because, at a low* H*_0_, the applied** H**_r_(*t*) cannot provide a strong enough magnetic torque for the pH-RPNR to resist the fluid viscous drag force, making it often rotate with a lower *f* than that of **H**_r_(*t*) (*ω*_r_ < *Ω*_r_). With the increasing *H*_0_, the magnetic torque exerted on the pH-RPNR is enhanced for it to resist the viscous drag, and the *v* of the pH-RPNR increases accordingly. When *H*_0_ was over a critical value (*H*_c_, *H*_c_ = 15 mT here), the pH-RPNR rotated synchronously with the applied** H**_r_(*t*) and moved in a stable *v* with the increasing *H*_0_. On the other hand, if the *f* was increased from 1 to 20 Hz but kept the *H*_0_ at 25 mT, the *v* of the pH-RPNR increased at first and then decreased with the increasing *f*, as shown in Fig. 2d. The maximum *v* was found to be 5.4 μm s^−1^ at the step-out frequency (${f}_{\mathrm{c}}$) of 8 Hz, after which the rotation of the pH-RPNR became unstable and asynchronous with the applied** H**_r_(*t*), and thus the *v* gradually decreased with the increasing *f* [54].

When a precessing magnetic field (**H**_w_(*t*)) [24] was applied, the pH-RPNR can move in a “walking” mode, in which two ends of the pH-RPNR rotating around the precession axis can act as its “feet” to alternatively touch the substrate, making the pH-RPNR “walk” smoothly on the substrate (Fig. S2a and Video S1). Similar to the “rolling” pH-RPNR (Fig. 2a-d), the “walking” pH-RPNR also employs the non-slip boundary-induced symmetry-breaking mechanism in their translational motions (Fig. S2a) and shows controllable directions and velocity depending on the precessing direction, *H*_0_ and *f* of the **H**_w_(*t*), respectively (Fig. S2b-d). The notable difference was found to be that the “walking” pH-RPNR showed a lower *H*_c_ (10 mT) and a higher *f*_c_ (16 Hz) than the “rolling” pH-RPNR (*H*_c_ = 15 mT, *f*_c_ = 8 Hz), and thus it had a higher maximum *v* (16.7 μm s^−1^) than the “rolling” pH-RPNR (*v* = 5.4 μm s^−1^). This difference can be attributed to the less moment of inertia in the “walking” mode (Fig. S2a), leading to the less energy required for the “walking” pH-RPNR to rotate and move in the liquid medium.

The pH-RPNRs have a periodic organization in structures, and thus they have a photonic bandgap and show visual structural colors (Fig. 2e). In addition, due to rich carboxyl groups in the hydrogel shell, their structural colors are highly sensitive to local pH changes (Fig. 2e). When local pH increases, carboxyl groups in the poly(AA-co-HEA) hydrogel shell of the pH-RPNR deprotonate into carboxylates, resulting in the swelling of the hydrogel shell due to the higher solubility of the latter [18]. This, in turn, makes the interparticle distance *d*, namely, the lattice spacing of the encapsulated photonic nanochain, become larger, leading to a red shift of the diffracted color of pH-RPNRs according to Bragg’s law [62, 63]:

2 $$\lambda =2nd\; \mathrm{sin}\theta $$

where $\lambda$ is the diffraction wavelength, $n$ is the relative refractive index, and $\theta$ is the angle between the incident light and the short axis of the pH-RPNR. In contrast, carboxylates in the poly(AA-co-HEA) hydrogel scaffold protonate into carboxyl groups when the surrounding pH decreases, resulting in the shrinkage of the hydrogel scaffold and the blue shift of the diffracted color of the pH-RPNR. To test the pH responses of the pH-RPNR, we first observed the length variation of a typical pH-RPNR at different pH (Fig. 2f). When a pH 7.4 buffer solution was added, the length of a horizontally-aligned pH-RPNR in a pH 4.4 buffer solution increased from 9.1 to 11.7 μm, reflecting its high deformation capability (elongation rate, 28.6%) in response to pH changes. With the elongation of the pH-RPNR, its structural color would change because of the increased *d* according to Bragg’s law (Eq. 2). To verify this, a drop of the pH 7.4 buffer solution was added at the right side (~ 4 mm away) of a vertically-reoriented pH-RPNR in pH 4.4 buffer solution (blue spot in Fig. 2g). With the diffusion of the pH 7.4 buffer solution, the pH-RPNR gradually changes its structural color from blue to green, yellow and red within 4.7 s (Fig. 2g). It is noted that the pH-RPNR had a high resolution of ~ 200 nm in pH sensing, which may pave the way for it to perform targeted on-the-fly pH detection in many hard-to-reach narrow spaces when combined with its magnetic propulsion.

### Swarming Motions and pH-Responsive Structural Colors

**Fig. 3:**
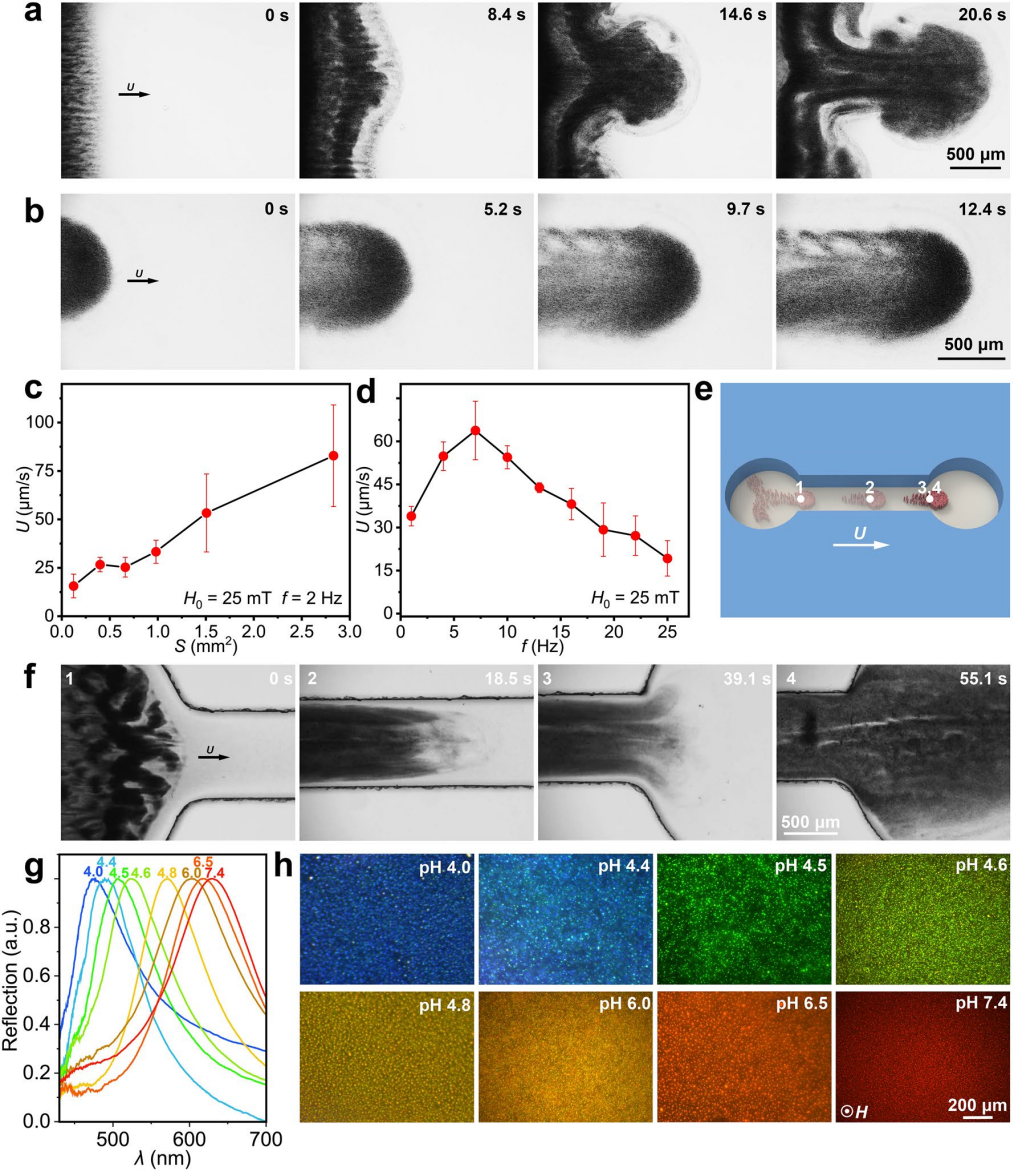
Collective motions and pH-responsive structural colors of swarming pH-RPNRs. **a** Time-lapse microscopic images demonstrating the formation of a mushroom-cloud-like swarm of the pH-RPNRs. **b** Time-lapse microscopic images depicting the collective motion of swarming pH-RPNRs. Collective velocity (*U*) of the swarming pH-RPNRs as a function of **c** the swarm size (*S*) and **d** *f*, respectively. **e** Schematic illustration and **f** time-lapse microscopic images of swarming pH-RPNRs passing through a microchannel. Numbers 1–4 in **e** denote the real-time positions of the pH-RPNR swarm in the microchannel when passing through it (**f**). **g** Reflection spectra and **h** dark-field optical microscopic images of the pH-RPNRs at different pH values

Except for the high-resolution sensing at the single-sensor level, rapid area-covering mapping of environmental physicochemical conditions by a group of nanorobots is also highly desired. Fortunately, similar to swarming organisms in nature, the pH-RPNRs can self-organize into microswarms under magnetic actuation when their local number density is increased. As shown in Fig. 3a and Video S2, when a rotating **H**_r_(*t*) with an *H*_0_ of 25 mT and an *f* of 3 Hz is applied, a stripe-like cluster of the gathered pH-RPNRs starts to propagate rightward, and as the frontline becomes unstable, it gradually transforms into a mushroom-cloud-like swarm. This phenomenon bears a resemblance to the formation of the mushroom-cloud-like flow pattern under Rayleigh–Taylor instability when a fluid jet intrudes into another fluid with similar density [64]. The swarming mechanism can be attributed to the hydrodynamic coupling between neighboring rolling pH-RPNRs [47]. Specifically, when a rotating **H**_r_(*t*) is applied, the magnetic pH-RPNR behaves as a roller that produces a small local vortex with a flow velocity decaying as *r*^−2^, where *r* is the distance to its rotation axis. In this way, neighboring rollers are attracted and advected by one another via hydrodynamic coupling and further gather into large compact rolling swarms rotating around their center of mass and translating along the substrate [47]. Due to the swarming behaviors, the pH-RPNRs show a striking collective effect, with which they move at a high collective velocity (*U*) of 86.5 μm s^−1^, about 50 times higher than single pH-RPNRs (1.8 μm s^−1^) at the same condition (Fig. 3b and Video S3). Moreover, the swarm is stable, as evidenced by the negligible changes in group structure when repeatedly activated and stopped or during prolonged movement (Fig. S3 and Video S4).

By adjusting the concentration of the pH-RPNRs in the aqueous medium, they, under an **H**_r_(*t*), can form into microswarms of tens of times different in size. Fig. S4 shows four typical microswarms with different areas (*S*) from 0.1 to 1.2 mm^2^. Further, we found that the *U* of the microswarms depends strongly on their *S*. As shown in Fig. 3c, under the fixed magnetic field parameters (*H*_0_ = 25 mT, *f* = 2 Hz), the *U* of the microswarm rises from 15.6 to 82.9 μm s^−1^ when its* S* increases from 0.1 to 2.8 mm^2^. The increasing *U* with *S* can be explained by the enhanced hydrodynamic coupling among neighboring rolling pH-RPNRs as the *S* increases. The collective velocity* U* of the swarming pH-RPNRs shows a positive linear correlation with *S* following *U* = *αSv*, where *α* was determined to be 24.9 in our experiments. In addition, the test of *f*-dependent velocity revealed that the microswarm (*S* =  ~ 2 mm^2^) shared a similar step-out frequency (*f*_c_ = 7 Hz) to single pH-RPNRs (*f*_c_ = 8 Hz) (Fig. 3d). When the rotating **H**_r_(*t*) was substituted with a precessing **H**_w_(*t*), the pH-RPNRs could also form into swarms while collectively moving in a "walking" mode. However, the formed swarms were relatively small in size (*S* =  ~ 0.28 mm^2^) due to the weaker produced flow field, as compared to those formed under the **H**_r_(*t*) with other conditions unchanged (*S* =  ~ 0.78 mm^2^), as shown in Fig. S5 and Video S5.

The pH-RPNRs can also swarm in a confined microchannel (Fig. 3e). When an **H**_r_(*t*) (*H*_0_ = 25 mT and *f* = 2 Hz) was applied, the pH-RPNRs in the left pool were activated and moved forward to the inlet of a narrow canal. In this process, the pH-RPNRs near the pool wall would move along it and then funnel into the open end of the narrow canal. As a result, they were condensed into a ribbon-like microswarm to fit through the narrow canal, revealing their exceptional environmental adaptability. Interestingly, the ribbon-like microswarm can traverse a 5 mm-long canal within 39.1 s, suggesting that it has a *U* of 127.9 μm s^−1^, which is much higher than the maximum *U* of those in open spaces with any *S* (82.9 μm s^−1^ at *f* = 2 Hz) (0–39.1 s in Fig. 3f and Video S6). This phenomenon can be rationalized by the fact that the microswarm can be regarded as a liquid stream, and its *U* fulfills the equation evaluating the flow velocity (*u*, corresponding to *U*) at the channel’s entry and exit when the volumetric flow rates are assumed identical [65], $u=Q/A$, where *Q* is the volumetric flow rate in the channel, and *A* is the cross-sectional area of the channel. Therefore, the decrease in *A* will result in an increase in *u*, corresponding to the increased *U* when the liquid-like microswarm flows from a large open pool into the narrow canal. Additionally, after fleeing through the narrow exit, the microswarm immediately expands to cover the other open pool on the right in a brief amount of time (39.1–55.1 s in Fig. 3f and Video S6). This quick covering behavior may facilitate rapid deployment of the pH-RPNRs over a sizable area to acquire pH information there.

Similar to the single pH-RPNRs, swarming pH-RPNRs also show different structural colors in response to pH changes. Because of the increased concentration, we are able to directly measure the reflection spectra of swarming pH-RPNRs with a high wavelength resolution. When the pH of the aqueous medium with suspended pH-RPNRs increases from 4.0 to 7.4, the diffraction peak ${(\lambda }_{\mathrm{max}})$ of the pH-RPNRs changes from 476 to 627 nm, corresponding to a diffraction-peak shift of up to 151 nm (Fig. 3g). This large shift in diffraction peaks indicates that the pH-RPNRs can change their structural colors over a wide range. As shown in Fig. 3h, their structural color can vary from dark blue to bright red with the pH changes, almost covering the whole visible spectrum. More than that, each bright dot in Fig. 3h represents a pH-RPNR reorientated perpendicular to the imaging planes, and different pH-RPNRs respond to local pH independently, which means that swarming pH-RPNRs can be used to perform microenvironmental pH-mapping tasks in high spatial resolution (~ 200 nm).

By adjusting the crosslinking degree of the pH-RPNRs, the color-change range and ${\lambda }_{max}$-shifting range (${\Delta \lambda }_{max}$) can be modulated. When the crosslinking degree of the pH-RPNRs increases from 3 to 6%, the ${\Delta \lambda }_{max}$ decreases from 122 to 30 nm in a pH range from 3.6 to 6.0, respectively (Fig. S6). This is because an increase in crosslinking degree makes the pH-RPNR stiffer and less sensitive to pH changes. Although pH-RPNRs require a static magnetic field to regulate the orientation for diffracting bright structural colors, the strength of the magnetic field showed no influence on their structural color (Fig. S7). These results indicate that the pH-RPNRs have a reliable and high-resolution pH-sensing capability.

### Targeted On-the-Fly pH Mapping

**Fig. 4:**
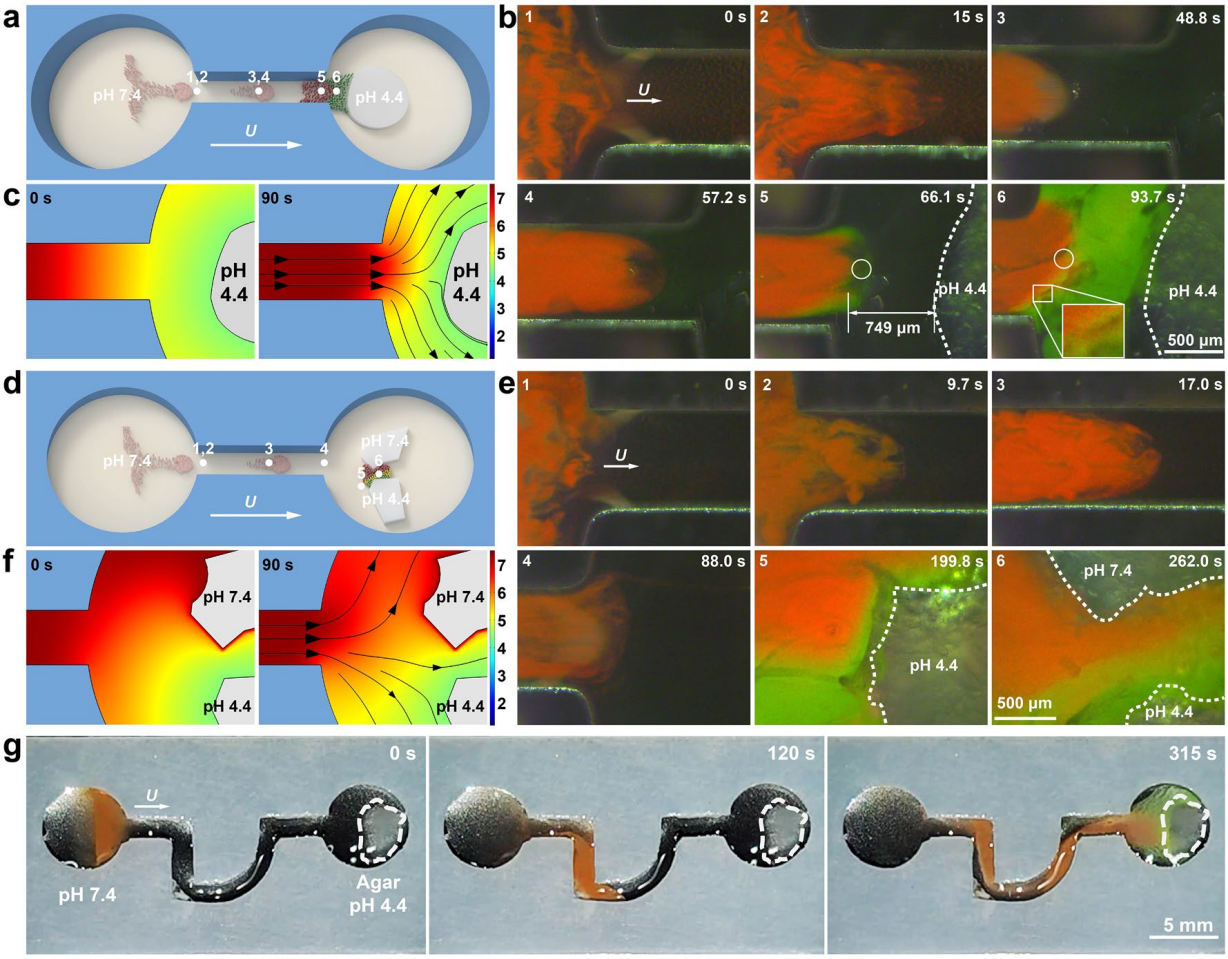
Targeted on-the-fly pH mapping by swarming pH-RPNRs. **a** Schematic illustration and **b** time-lapse dark-field microscopic images of the swarming pH-RPNRs when performing targeted on-the-fly pH mapping by collectively moving from a microwell with pH 7.4 toward an agar gel with pH 4.4. **c** Simulated time-dependent pH map around the agar gel. **d** Schematic illustration and **e** time-lapse dark-field microscopic images of the swarming pH-RPNRs when collectively moving toward two ager gels with pH 7.4 and pH 4.4. **f** Simulated time-dependent pH map around two agar gels. Numbers 1–6 in (**a** and **d**) denote the real-time positions of the pH-RPNR swarm in the microchannel when performing the pH-mapping tasks (**b** and **e**). **g** Digital photographs demonstrating that the swarming pH-RPNRs can move along a tortuous route to perform targeted on-the-fly pH mapping at the macroscopic scale with naked-eye detectable structural color changes

Benefitting from their collective motions and pH-responsive structural colors, swarming pH-RPNRs are envisioned to be navigated to perform targeted on-the-fly pH sensing and mapping toward a specific target. The targeted on-the-fly pH mapping was investigated in a microfluidic chip with two open pools connected by a narrow canal, in which a piece of low-pH agar gel was placed in the right pool as the target (Fig. 4a). When actuated by the **H**_r_(*t*) (*H*_*0*_ = 25 mT and *f* = 2 Hz), swarming pH-RPNRs in the left pool passed through the narrow canal and moved toward the target (pH 4.4 agar gel) (Fig. 4b and Video S7). Before reaching the right pool, the swarming pH-RPNRs exhibited a bright red structural color, reflecting the pH of the aqueous medium (pH 7.4 buffer) (0–57.2 s in Fig. 4b). Once reaching the right pool, the pH-RPNRs at the front of the microswarms changed their structural color from red to green when they were about 750 μm away from the targeted agar gel (66.1 s in Fig. 4b). This is because of the continuous diffusion of H^+^ out of the agar gel. When the swarming pH-RPNRs finally reached the targeted agar gel and covered the space around it, a clear pH map is immediately depicted (93.7 s in Fig. 4b), in which the region near the targeted agar has a pH of 4.5 (in green color) and that away from it is 7.4 (in red color), and a pH gradient between these two regions is clearly observed in the color-transition band (the inset in Fig. 4b at 93.7 s). This pH map agrees well with the simulated result based on H^+^ diffusion (Fig. 4c). It is important to note that the pH-RPNR microswarm is analogous to a fluid jet that can push the diffused H^+^ backward, thereby reversing the diffusion of H^+^ (Fig. 4c). This anti-diffusion effect can be verified by the fact that the pH-RPNRs at a fixed position (white circles in Fig. 4b at 66.1 and 93.7 s) in the right pool experience color changes from green to red (i.e., pH increase) as the pH-RPNRs continuously flow rightward, and also that the green-color front of the swarming pH-RPNRs rapidly propagates leftward (corresponding to the diffusion direction of H^+^) when they were stopped (Fig. S8 and Video S7). This result suggests that swarming pH-RPNRs may act as a self-reporting micropump.

Furthermore, more challenging testing conditions were established to assess the robustness of the targeted on-the-fly pH mapping capability. The experiment was conducted by placing two agar gels with significant pH differences in the right pool (Fig. 4d) but keeping other conditions the same as those shown in Fig. 4b. At first, similar collective motions toward the targets and structural color-based pH mapping behaviors near the pH 4.4 agar were observed for the swarming pH-RPNRs (0–199.8 s in Fig. 4e). Astonishingly, two agar targets with different pH of 4.4 and 7.4 were perfectly distinguished by the respective distinct green and red structural colors of the pH-RPNRs around each target. The pH gradient in the space between the two agars was visualized by a red-yellow-green color-transition band (262.0 s in Fig. 4e and Video S8), agreeing well with the simulated pH map (Fig. 4f). These results further verify the robust targeted on-the-fly pH mapping capability of the swarming pH-RPNRs.

Due to the magnetic field-navigated collective motions and bright pH-responsive structural colors, the pH-RPNRs can swarm at the macroscopic level along a tortuous route and realize targeted on-the-fly pH mapping, even detectable by the naked eye (Fig. 4g and Video S9). When navigated by an **H**_r_(*t*), the swarming pH-RPNRs can travel in a tortuous canal with multiple turns, and successfully arrive at the right pool with a target of a pH 4.4 agar gel (Fig. 4g). Once approaching the target, they detected the pH changes there and changed their structural color from light red to light green in response, with which the pH near the target can be directly read out by the naked eye (315 s in Fig. 4g). Although their structural color is not as bright as that under the dark-field macroscopic condition due to the reduced contrast as a result of the diffuse reflection of ambient light, it still has a distinct pH-dependent appearance and exhibits a similar experimental outcome as depicted in Fig. 4b. This suggests that the swarming pH-RPNRs developed here may largely alleviate instrumental requirements in sensing compared to the developed single sensing MNRs.

The microscopic and macroscopic sensing performance indicates that the swarming pH-RPNRs can actively patrol in complex environments utilizing their navigatable collective motions and then identify unknown targets by mapping local abnormal pH conditions via responsive structural colors. In contrast, the immotile nanosensors (e.g., passive fluorescent nanoprobes) cannot achieve motile-targeting sensing. On the other hand, the previously reported motile micro/nanosensors are mainly based on chemically-propelled MNRs with random motions and additional (toxic) fuels, and can only generate analytical signals (e.g., speed and fluorescence changes) after irreversibly binding with analytes. Thus, they show low targetability, poor biocompatibility, chemical-fuel interference, and poor reversibility (one-time use only) [38, 39]. More importantly, they have no swarming navigation control and can only perform sensing at a single-robot level, suffering from limited area-covering mapping capability, indirect readout (sophisticated analytical instruments), and poor reproducibility (individual differences).

### Swarming Temperature- and Glucose-Responsive Photonic Nanorobots

**Fig. 5:**
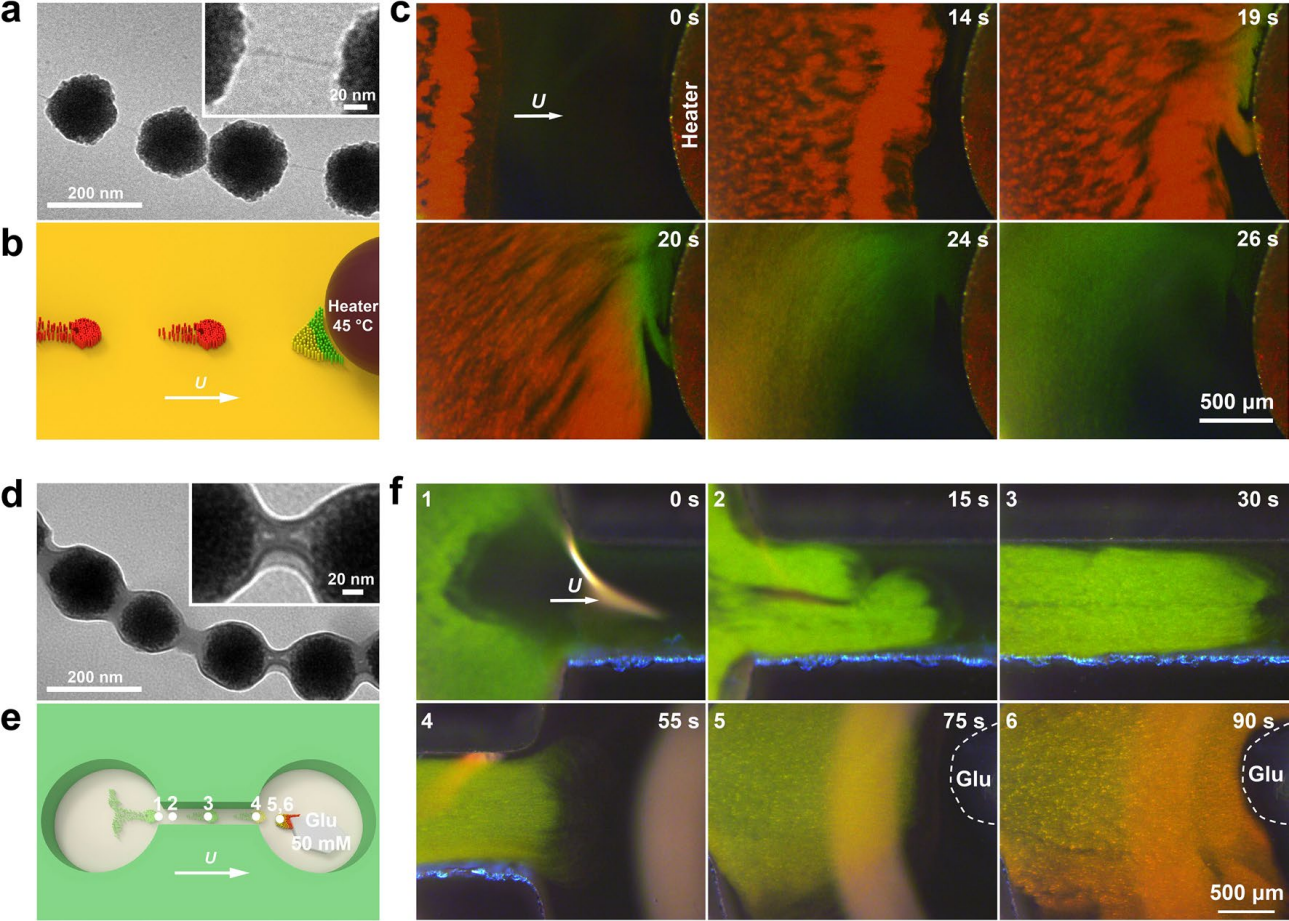
Swarming temperature- and glucose-responsive nanorobots and their targeted on-the-fly mapping. **a** TEM images of temperature-responsive photonic nanorobots (T-RPNRs) with a Fe_3_O_4_@poly(NIPAM-co-NHMA) core–shell nanochain-like structure. **b** Schematic illustration and **c** time-lapse dark-field microscopic images depicting swarming motions of swarming T-RPNRs toward a heater and their structural color change when the heater is heated to 45 °C. **d** TEM images of glucose-responsive photonic nanorobots (G-RPNRs) with a Fe_3_O_4_@poly(AAPBA-co-HEAA) core–shell nanochain-like structure. **e** Schematic illustration and **f** time-lapse dark-field microscopic images depicting the targeted on-the-fly glucose sensing and mapping of swarming G-RPNRs toward an agar gel with 50 mM glucose (Glu). Numbers 1–6 in **e** denote the real-time positions of the G-RPNR swarm in the microchannel when performing the glucose-mapping task (**f**)

By simply using the monomers with different functional groups in the preparation, different RPNRs can be developed to realize targeted on-the-fly mapping of different physicochemical signals. For example, when the monomers of AA and HEA were substituted with NIPAM and NHMA, temperature-responsive photonic nanorobots (T-RPNRs) composed of Fe_3_O_4_ NPs and poly(NIPAM-co-NHMA) hydrogel can be obtained. Similar to the pH-RPNRs (Fig. S1e), the T-RPNRs also have a core–shell nanochain structure but with a much thinner hydrogel shell (~3 nm, Figs. 5a and S9a, b). With the increasing temperature from 38 to 45 °C, the diffraction peak ${\lambda }_{\mathrm{max}}$ of the T-RPNRs changes from 627 to 553 nm (Fig. S9c), corresponding to the structural color change from red to green (Fig. S9d). When actuated by an **H**_r_(*t*), the T-RPNRs could collectively move toward a heat source (i.e., a heating rod) and detect the temperature there (Fig. 5b). When swarming T-RPNRs arrived at the target area, the heating rod was turned on and heated the aqueous medium from room temperature to 45 °C. As a response, the red T-RPNRs near the heating source changed their structural color to green due to the shrinkage of the hydrogel shell with its phase transition at a high temperature [19, 66, 67], visualizing the local temperature change and distribution (Fig. 5c and Video S10).

Similarly, when the monomers of AAPBA and HEAA were used in the preparation, the glucose-responsive photonic nanorobots (G-RPNRs) consisting of a Fe_3_O_4_ nanochain enveloped in a poly(AAPBA-co-HEAA) hydrogel shell are generated (Figs. 5d and S10a, b). When the glucose concentration *C*_Glu_ increases from 0 to 50 mM, the diffraction peak ${\lambda }_{\mathrm{max}}$ of the G-RPNRs changes from 494 to 608 nm (Fig. S10c), and their structural color changes from green to red (Fig. S10d). When actuated by an **H**_r_(*t*), they can perform targeted on-the-fly mapping toward a local glucose source in a microfluidic channel (Fig. 5e). As shown in Fig. 5f and Video S11, the swarming G-RPNRs leave from one end of the channel and reach the target area (a piece of agar gel loaded with 50 mM glucose) within 90 s. When perceiving glucose molecules by forming a charged complex with AAPBA in the hydrogel shell [20], the G-RPNRs, as the hydrogel shell expands, gradually shift their structural color from green to orange, mapping the local distribution of glucose. These results indicate the versatility of the swarming RPNRs to detect and map different physicochemical conditions in microenvironments, such as pH, temperature, and glucose concentration.

### Mapping-Guided Photothermal Treatment

**Fig. 6:**
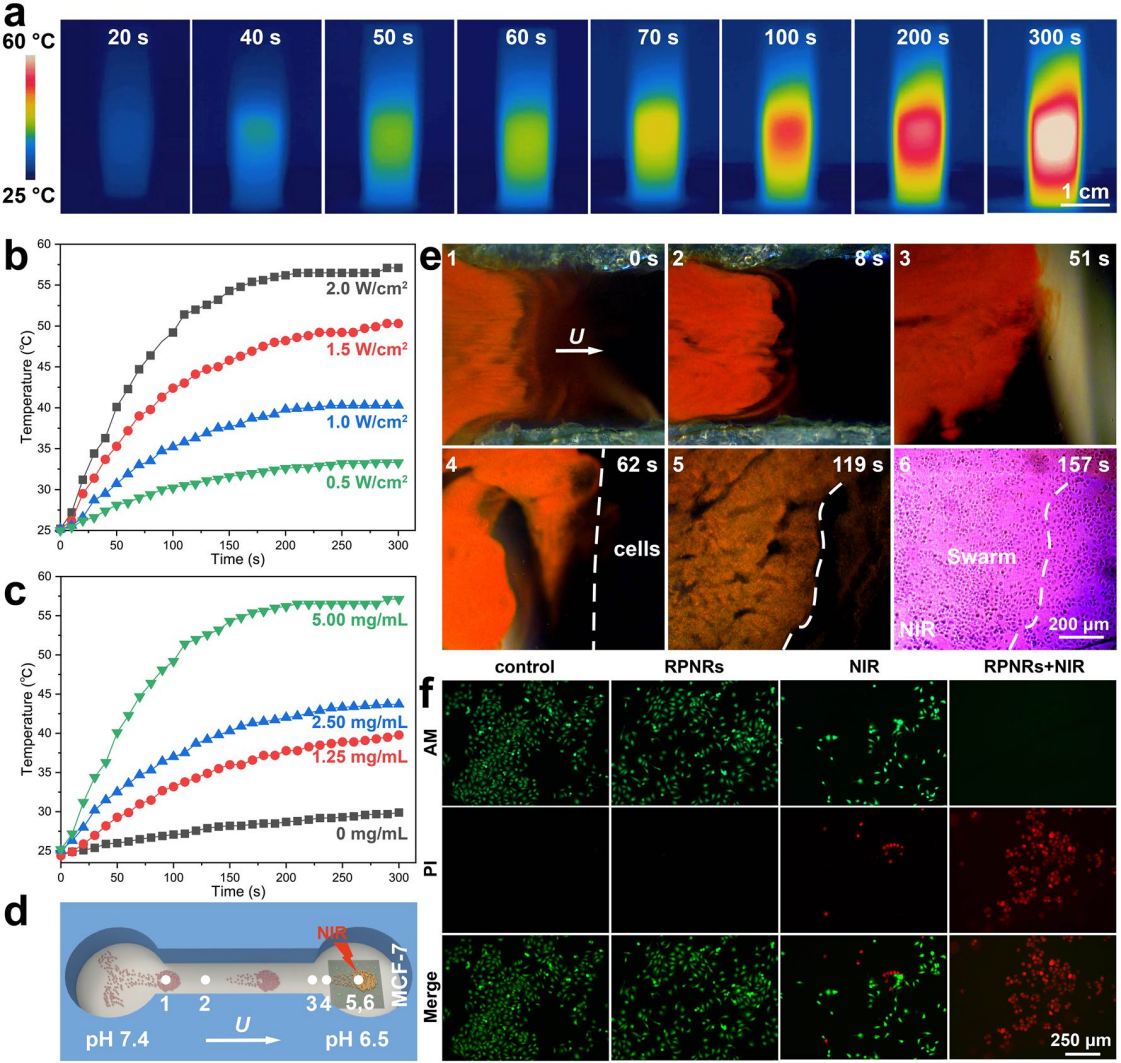
Mapping-guided photothermal therapy by swarming pH-RPNRs. **a** Time-lapse thermal images of the pH-RPNRs when irradiated by a near-infrared (NIR) laser (808 nm, 2.0 W cm^−2^). Photothermal heating curves of the aqueous medium with the pH-RPNRs at **b** different power densities (*I*) of NIR laser and **c** different pH-RPNR concentrations (*C*_n_). **d** Schematic illustration and **e** time-lapse dark-field microscopic images depicting the rapid covering of MCF-7 tumor cells and subsequent mapping-guided photothermal therapy by the swarming pH-RPNRs. **f** Live/dead staining results of MCF-7 tumor cells after different treatments. The green and red fluorescence indicate the live and dead cells, respectively

Photothermal treatment (PTT), which employs photosensitizers to induce local hyperthermia by converting light energy into heat, has been considered as a promising treatment for many diseases due to its unique advantages, such as excellent selectivity, minimal invasiveness, and limited side effects [68]. To investigate the potential of swarming RPNRs in PTT, the pH-RPNRs were selected as an example, and their photothermal effect was tested at first. Owing to the photothermal conversion of the Fe_3_O_4_ component [56], when exposed to a NIR laser with a power density (*I*) of 2.0 W cm^−2^, the aqueous suspension of the pH-RPNRs (concentration *C*_n_, 5 mg mL^−1^) could be quickly heated up to ~ 60 °C within 300 s, as verified by the time-lapse thermal images shown in Fig. 6a. This temperature is 35 and 15 °C higher than the room temperature and the temperature required for PTT (42–45 °C) [69], respectively. The photothermal heating curves of the aqueous medium with the pH-RPNRs (*C*_n_ = 5.00 mg mL^−1^) at a different *I* are shown in Fig. 6b. It can be seen that the temperature of the aqueous medium increases with the irradiation time at any *I*, and becomes stable after around 250 s. The maximum temperature (*T*_m_) was found to be decreasing from 57.1, 50.3, 40.3 to 33.3 °C with the decreasing *I* from 2.0, 1.5, 1.0 to 0.5 W cm^−2^, respectively. Similarly, the aqueous medium with different *C*_n_ also reaches a *T*_m_ after 250 s NIR irradiation (*I* = 2.0 W cm^−2^), and the *T*_m_ is 57.1, 43.7, and 39.8 °C when* C*_n_ decreased from 5.00, 2.50 to 1.25 mg mL^−1^, respectively, as demonstrated in Fig. 6c. The optimal conditions for the pH-RPNRs to perform PTT are found to be at a* C*_n_ of 2.50 mg mL^−1^ and an *I* of 2.0 W cm^−2^ as their *T*_m_ (43.7 °C) is in the PTT temperature range (42–45 °C) [69].

In addition to investigating the photothermal effect, the biocompatibility of pH-RPNRs has also been studied. A dose-dependent cytotoxicity test showed that MCF-7 cells had a high survival rate of close to 100% after incubation with pH-RPNRs of different *C*_n_ (0–2 mg mL^−1^) for 24 h, indicating their non-cytotoxicity (Fig. S11). Furthermore, when pH-RPNRs were suspended in a PBS buffer containing 10% FBS for 24 and 48 h, no agglomerations, morphology changes, or significant zeta potential variations were observed, suggesting their high stability in the biological medium (Fig. S12a-c). Additionally, hemolysis evaluation showed that pH-RPNRs exhibited a low hemolysis rate of 1.0% and 2.9% at *C*_n_ of 2.5 and 5 mg mL^−1^, respectively, suggesting that they have a negligible influence on the damage of red blood cells (Fig. S12d). The high biocompatibility of pH-RPNRs may be attributed to their low chemical toxicity, soft hydrogel surface, and strong electrostatic repulsion to cells [70–72].

Considering the low pH of tumor microenvironment (5.8–7.2) [6], as well as multiple integrated functions of biocompatible swarming pH-RPNRs including magnetic propulsion, visual pH mapping, and photothermal conversion, they are envisioned to realize motile-targeting mapping-guided photothermal treatment. Specifically, they may rapidly move toward and cover a target area under magnetic propulsion, then label a low-pH tumor lesion via visual mapping, and further guide the external NIR irradiation to achieve localized photothermal treatment on demand. To verify this prediction, we used MCF-7 cells grown on a glass slide with a pH of 6.5 as the simulated tumor lesion, and the MCF-7 cells were put in the right pool of a microfluidic channel to serve as an unknown target (Fig. 6d). As shown in Fig. 6e and Video S12, the swarming pH-RPNRs in the left pool can move toward and cover the target area (right pool) after passing through a narrow canal under the **H**_r_(*t*) (0–62 s in Fig. 6e). After covering the target area, the structural color of pH-RPNRs shifted from red to orange due to the low pH there. Even though no tumor cells could be directly observed under dark-field microscopy, the simulated tumor site was recognized based on the translation of the local pH condition to visual optical signals (119 s in Fig. 6e). When receiving this visual orange signal, a beam of NIR light was locally applied to activate the photothermal conversion of the swarming pH-RPNRs to kill the tumor cells (MCF-7 cells) (157 s in Fig. 6e).

After the PTT treatment for 5 min, all MCF-7 cells were dead (cell death rate, ~ 100%), as confirmed by the fluorescent microscopic images of dead (red) and alive (green) MCF-7 cells (Fig. 6f). In contrast, no cell death was observed in the control group (without pH-RPNRs and NIR irradiation) and the pH-RPNRs-only group, and the cell death rate in the NIR-only group is low as 14.6%, indicating that NIR irradiation alone has a minimal capacity to cause tumor-cell apoptosis. These results indicate that the tumor-cell apoptosis is mainly induced by the photothermal heating of the pH-RPNRs. Besides the low-pH condition, temperature and glucose levels around tumor sites are also different from those of normal tissues, and can also be exploited as physiological indicators of the tumor microenvironment [2]. Thus, the swarming RPNRs with temperature or glucose responsiveness (Fig. 5) also have great potential in motile-targeting mapping-guided photothermal tumor treatment because of their integrated functions of the targeted on-the-fly temperature/glucose mapping and photothermal conversion.

Compared to the reported magnetic MNRs [24, 46], the swarming RPNRs have a periodic arrangement in structures and show intelligent responses to surrounding physicochemical changes, thereby enabling emerging applications in (bio)sensing and disease treatment. In (bio)sensing, the swarming RPNRs provide a robust active motile-targeting strategy for microenvironmental mapping because they can actively navigate in complex environments and collectively map out local abnormal physicochemical conditions (e.g., pH, temperature, or glucose concentration) on the fly via their bright responsive structural colors. Through swarming behaviors, the RPNRs have built linkages between nano-, micro-, and macro-sensing, with which they have a high spatial resolution at the individual level, and show high fault tolerance, rapid large-area mapping, and even naked-eye detectable structural colors at the swarm level. The visual mapping signals (structural colors) of the RPNRs are in the visible-light range. Thus, they can be directly used in *in-vitro* microfluidic analytical devices and *in-vivo* transparent or endoscopically accessible organs (e.g., eyeball, lung, esophagus, gastrointestinal tract, and bladder) [73, 74]. In addition, their responsive deformations and adjustable interparticle distances may also alter the signal from deep-penetration imaging technologies, such as ultrasound imaging (USI), computed tomography (CT), and magnetic resonance imaging (MRI). Therefore, they may also generate responsive USI, CT, and MRI signals when perceiving abnormal physicochemical conditions, realizing motile-targeting microenvironmental mapping in deep tissues [75, 76].

In the photothermal treatment, different from traditional immotile photothermal nanoagents [68], the developed swarming RPNRs can actively find and visualize an unknown tumor lesion via motile-targeting mapping to guide the external NIR light to initiate localized photothermal treatment. As the visual color-mapping signals of the swarming RPNRs can be captured and tracked by the external imaging apparatus, an automatic theranostic nanorobot system is expected to be realized in the future by developing computer programs to coordinate imaging apparatus, artificial intelligence navigation planners, magnetic-field generators, and external NIR source in the future [77]. On the other hand, due to the drug loading capacity of the hydrogel shell via different interactions (e.g., electrostatic, hydrophobic, and hydrogen-bond interactions), as well as their responsive drug release in response to local pH, temperature, or glucose concentration [4], the additional function of stimulus-triggered drug delivery may also be integrated to the RPNRs, enabling them to act as an intelligent multifunctional motile theranostic platform. For future *in-vivo* applications, the swarming RPNRs could be administered via various routes, such as topical, oral, intralesional, inhalation, or instillation administration. After administration, the nanorobots can be collected near the targeted area using a permanent magnet and then actuated to patrol that area under alternating magnetic fields. In case a superficial tumor site is detected via the microenvironmental mapping, the swarming RPNRs can be activated to penetrate the tumor tissue through narrow interstitial spaces for subsequent photothermal treatment. Alternatively, if no tumor is detected, they can be navigated to patrol other regions of interest.

## Conclusions

In summary, we have demonstrated that swarming RPNRs consisting of a nanochain of periodically arranged Fe_3_O_4_ NPs encapsulated in a responsive hydrogel shell can actively navigate in complex environments, visualize unknown targets on the fly, and guide localized photothermal treatment. The experimental results show that the swarming RPNRs show energetic collective motions with a maximum collective velocity of 127.9 μm s^−1^ when navigated by a rotating **H**_r_(*t*), have a wide structural color-shift range of about ~ 150 nm (from dark blue to bright red), and can reach the PPT temperature (~ 45 °C) within 300 s under NIR irradiation. They have been confirmed to actively pass through a narrow microchannel to rapidly cover a targeted microenvironment, then visualize unknown targets (e.g., tumor lesion) by mapping the abnormal condition (e.g., pH, temperature, or glucose concentration) there via their responsive structural colors, and finally induce tumor cell apoptosis on demand via mapping-guided localized photothermal treatment. This work may essentially promote the development and applications of intelligent motile nanosensors and nanotheranostics for cancer and inflammatory diseases.

### Supplementary Information

Below is the link to the electronic supplementary material.

Supplementary file1 (PDF 904 kb)

Supplementary file2 (MP4 7073 kb)

Supplementary file3 (MP4 2891 kb)

Supplementary file4 (MP4 3630 kb)

Supplementary file5 (MP4 3837 kb)

Supplementary file6 (MP4 4112 kb)

Supplementary file7 (MP4 2948 kb)

Supplementary file8 (MP4 6842 kb)

Supplementary file9 (MP4 12619 kb)

Supplementary file10 (MP4 4456 kb)

Supplementary file11 (MP4 3051 kb)

Supplementary file12 (MP4 4774 kb)

Supplementary file13 (MP4 7817 kb)
