# Supplementary material for: Swarming Responsive Photonic Nanorobots for Motile-Targeting Microenvironmental Mapping and Mapping-Guided Photothermal Treatment
Source: Nanomicro Lett. 2023 May 29;15:141. doi: 10.1007/s40820-023-01095-5 (PMC10226971; doi:10.1007/s40820-023-01095-5)
Supplement: Supplementary file 1 — Supplementary file1 (PDF 904 kb) [file 40820_2023_1095_MOESM1_ESM.pdf]

Supporting Information for

Swarming Responsive Photonic Nanorobots for Motile-Targeting Microenvironmental Mapping and Mapping-Guided Photothermal Treatment

Luolin Li[1], Zheng Yu[1], Jianfeng Liu[1], Manyi Yang[1], Gongpu Shi[1], Ziqi Feng[1], Wei Luo[1,*], Huiru Ma[3,4], Jianguo Guan[1,2], Fangzhi Mou[1,*]

[1]State Key Laboratory of Advanced Technology for Materials Synthesis and Processing, Wuhan University of Technology, Wuhan 430070, P. R. China

[2]School of Materials and Microelectronics, Wuhan University of Technology, Wuhan 430070, P. R. China

[3]School of Materials Science and Engineering, Wuhan University of Technology, Wuhan 430070, P. R. China

[4]School of Chemistry, Chemical Engineering and Life Science, Wuhan University of Technology, Wuhan 430070, P. R. China

*Corresponding authors. E-mail: moufz@whut.edu.cn (Fangzhi Mou); rowell@whut.edu.cn (Wei Luo)

Supplementary Figures

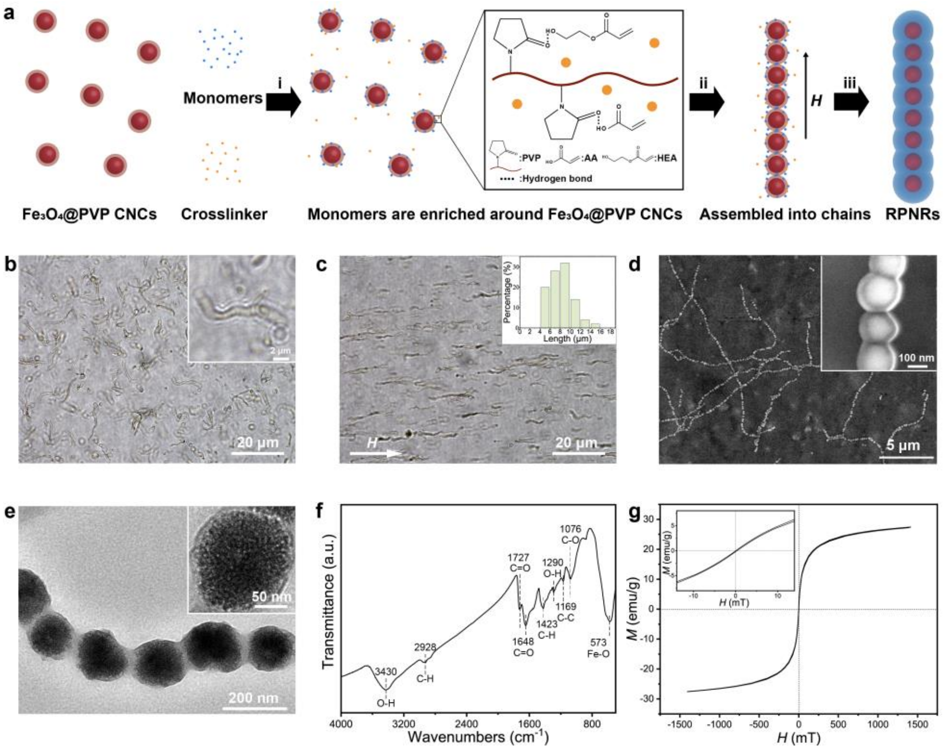

Fig. S1 Preparation and characterization of pH-RPNRs. a Schematic illustration of the preparation of pH-RPNRs. (i) Monomers concentrate around Fe_3O_4@polyvinylpyrrolidone nanoparticles (Fe_3O_4@PVP NPs); (ii) Self-assembly of

the Fe_3O_4@PVP NPs with the absorbed monomers into a nanoparticle chain under a static magnetic field (H); (iii) UV light-initiated gelation of the monomers and the formation of the responsive hydrogel shell on the nanoparticle chain. **b-g** Characterization of pH-RPNRs. Optical microscopic (**b, c**), SEM (**d**) and TEM (**e**) images, FT-IR spectrum (**f**), and magnetic hysteresis loop (**g**) of the Fe_3O_4@poly(AA-co-HEA) pH-RPNRs. Insets in b, d and e depict the corresponding highly-magnified images. The inset in c gives the histogram of the chain length distribution.

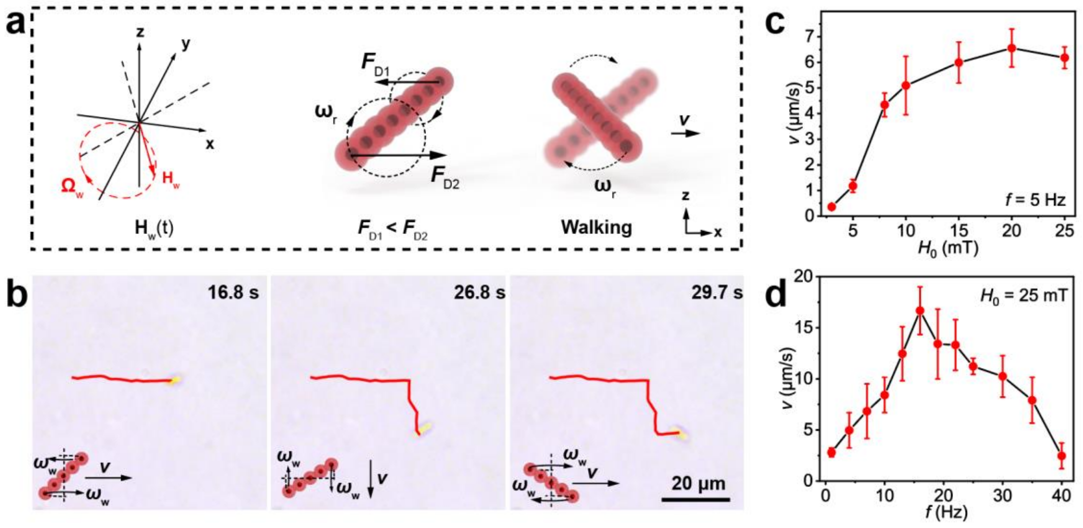

Fig. S2. The "walking" mode of pH-RPNRs. a Schematic illustration of a pH-RPNR moving in a "walking" mode under a precessing magnetic field ($\mathbf{H}_w(t)$). **b** Time-lapse microscopic images depicting a "walking" pH-RPNR moving in a zigzag trajectory (red curves) when navigated by the $\mathbf{H}_w(t)$. The velocity (v) of the "walking" pH-RPNRs as a function of the strength (H_0) (**c**) and frequency (f) (**d**) of $\mathbf{H}_w(t)$

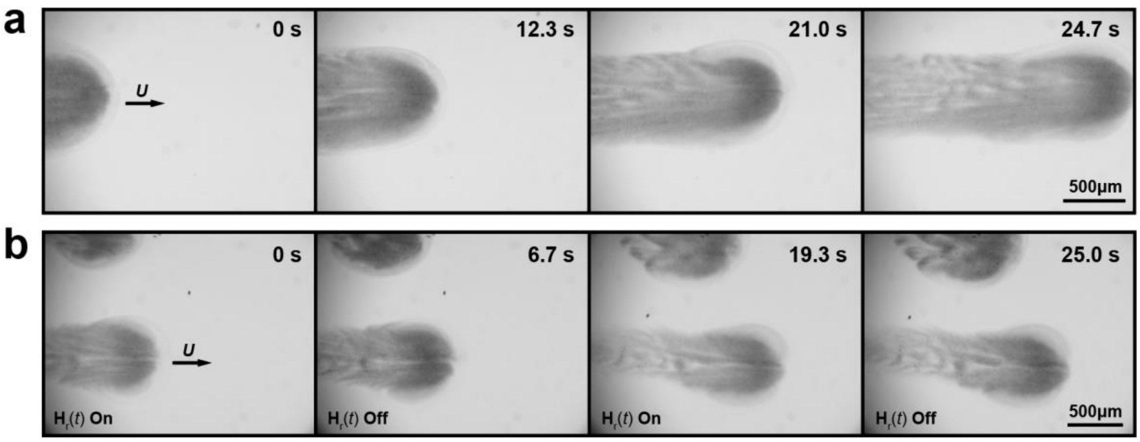

Fig. S3 Stability of pH-RPNR swarms. Time-lapse microscopic images depicting the group structure of swarming pH-RPNRs during prolonged movement (**a**) and when repeatedly activated and stopped by the $\mathbf{H}_r(t)$ (**b**)

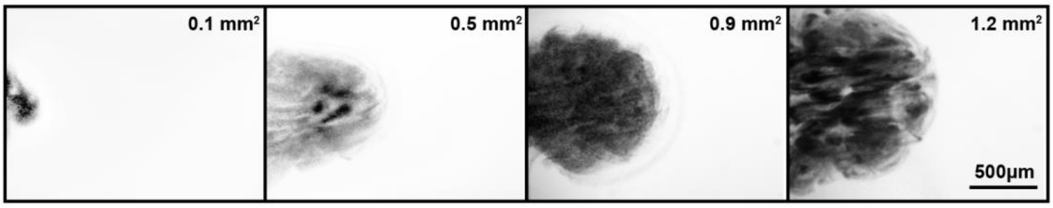

Fig. S4 pH-RPNR swarms with different sizes (*S*). Under the $\mathbf{H}_r(t)$, pH-RPNRs can form into microswarms of tens of times different in size by adjusting their concentration

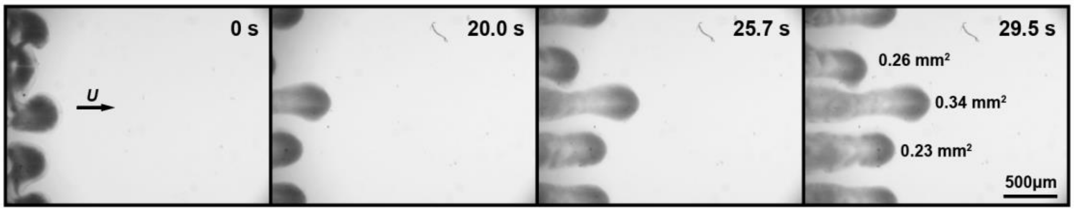

Fig. S5 Swarms of the "walking" pH-RPNRs. Time-lapse microscopic images show the sizes and collective motions of the swarms formed by "walking" pH-RPNRs under a precessing $\mathbf{H}_w(t)$

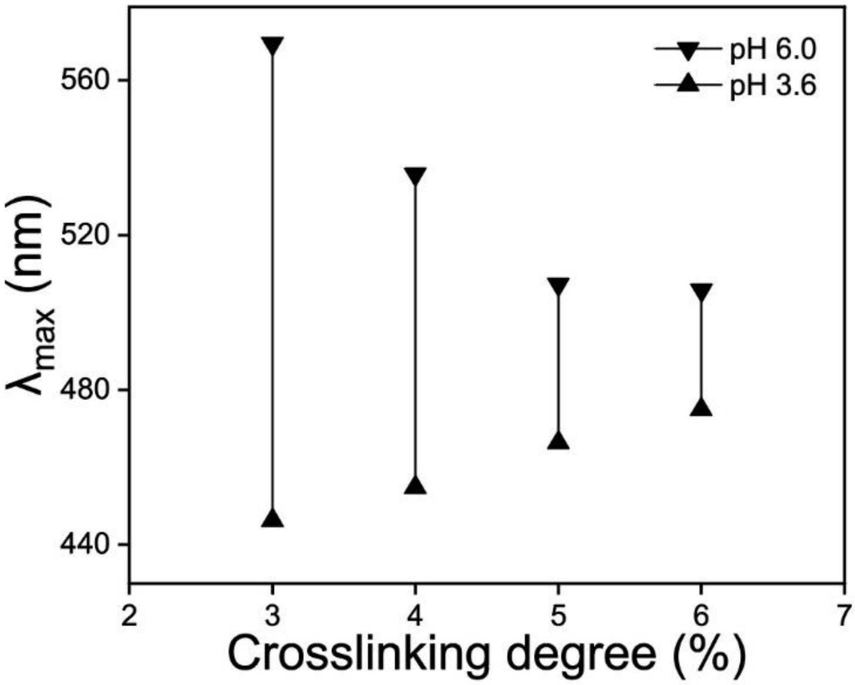

Fig. S6. Diffraction peaks (λ_{max}) of the pH-RPNRs with different crosslinking degrees. When the crosslinking degree of the pH-RPNRs increases from 3% to 6%, the color-change range and λ_{max}-shifting range ($\Delta\lambda_{max}$) decreases from 122 to 30 nm in a pH range from 3.6 to 6.0, respectively

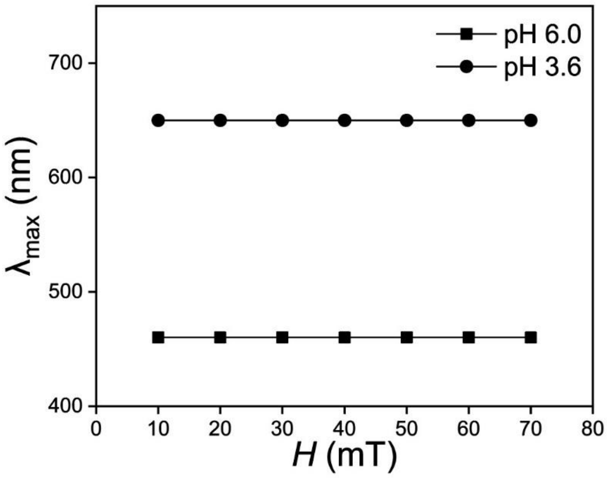

Fig. S7 Diffraction peak (λ_{max}) of pH-RPNRs at different strengths (H) of the applied static magnetic field. This result reveals that the H has negligible influence on λ_{max} of the pH-RPNRs.

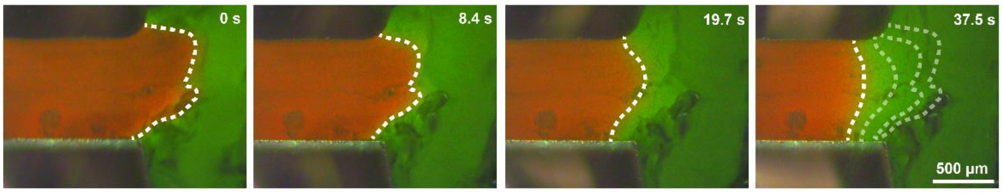

Fig. S8 Diffusion of H^+ visualized by the structural color change of pH-RPNRs. After the swarming pH-RPNRs are stopped, the green-color front rapidly propagates leftward in 37.5 s.

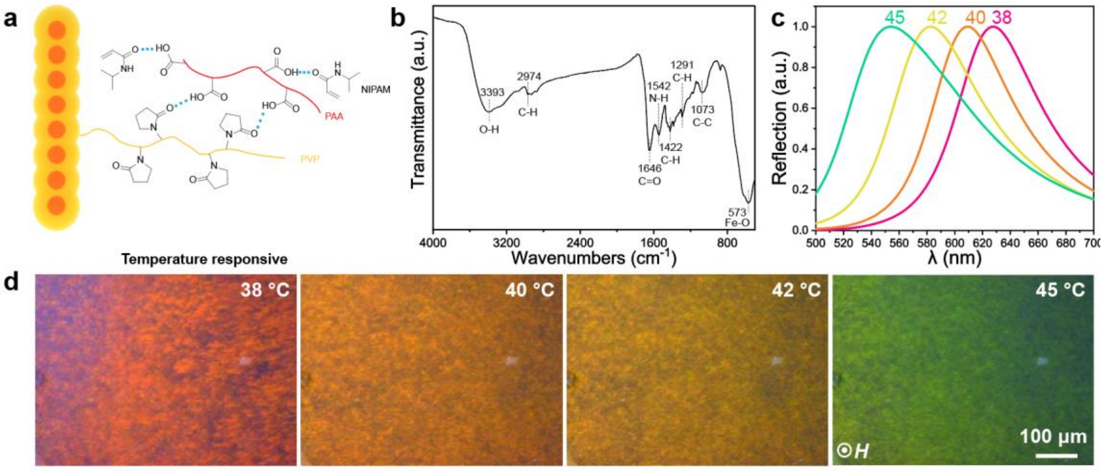

Fig. S9 Fe_3O_4@poly(NIPAM-co-NHMA) T-RPNRs. a Schematic illustration of the chemical composition of T-RPNRs. **b** FT-IR spectrum of T-RPNRs. Reflection spectra (**c**) and dark-field optical microscopic images (**d**) of the T-RPNRs at different temperatures

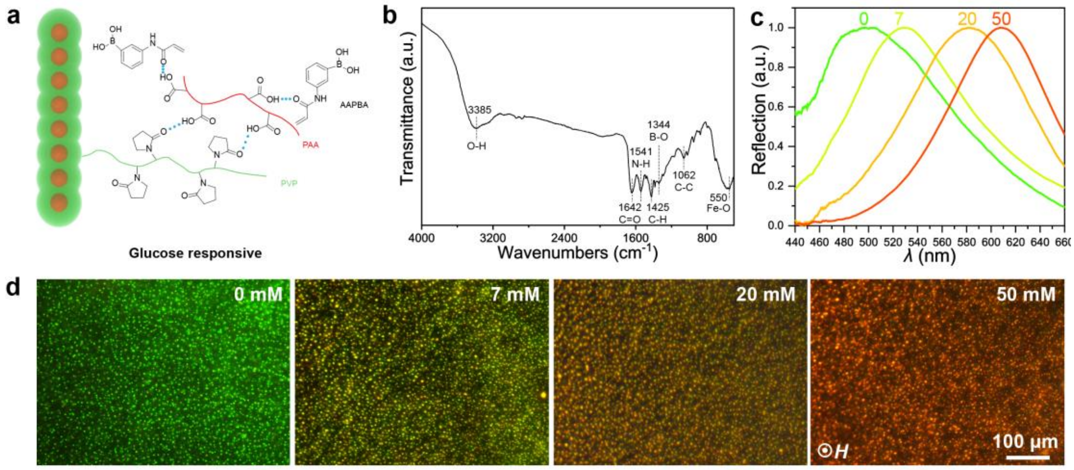

Fig. S10 Fe_3O_4@poly(AAPBA-co-HEAA) G-RPNRs. a Schematic illustration of the chemical composition of G-RPNRs. **b** FT-IR spectrum of G-RPNRs. Reflection spectra (**c**) and dark-field optical microscopic images (**d**) of the G-RPNRs at glucose concentration

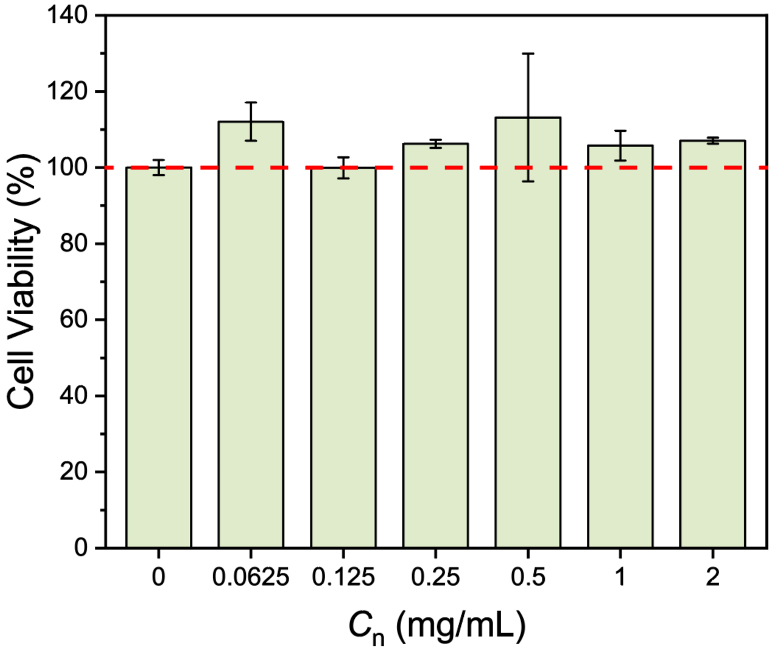

Fig. S11 MCF-7 Cell viability after co-incubation with pH-RPNRs of different concentrations (C_n) at 37 °C and 5% CO_2 for 24 h

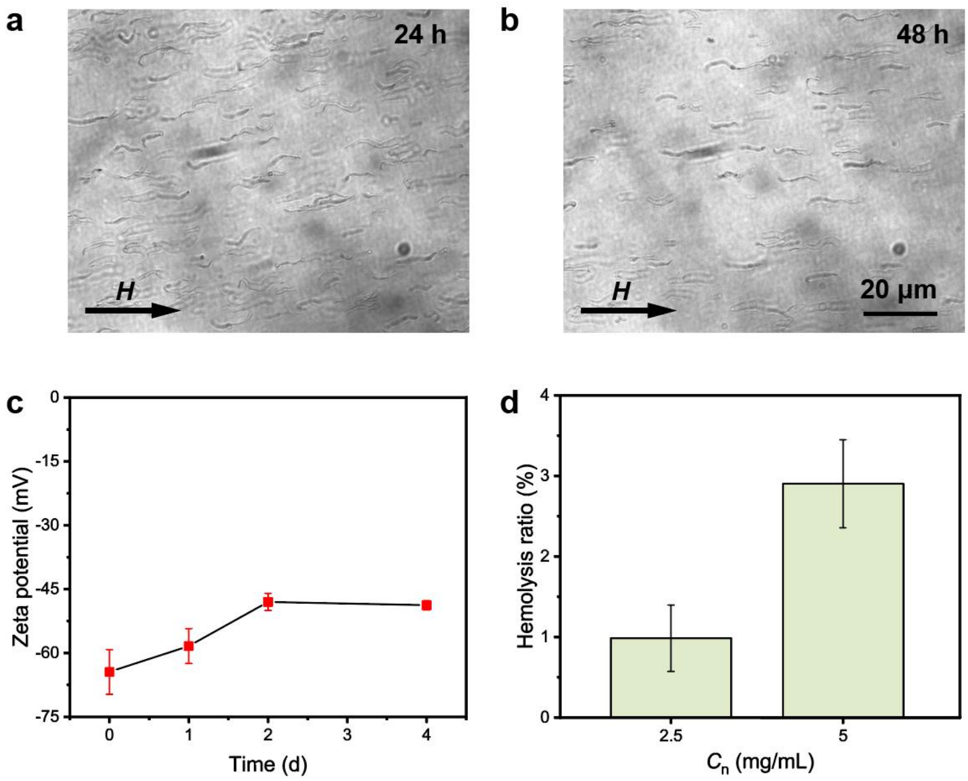

Fig. S12 Stability and hemocompatibility of pH-RPNRs. Optical microscopic images of pH-RPNRs after incubation in the PBS buffer containing 10% fetal bovine serum (FBS) for 24 (**a**) and 48 h (**b**). **c** Zeta potential of pH-RPNRs after incubation in the PBS buffer containing 10% FBS for different time. **d** Hemolysis rate of pH-RPNRs at different C_n.

Description of Supporting Videos

Video S1 A typical pH-RPNR moving in a predesigned trajectory in a "rolling" and "walking" mode when navigated by the $\mathbf{H}_r(t)$ and $\mathbf{H}_w(t)$, respectively.

Video S2 The formation of a mushroom-cloud-like swarm of pH-RPNRs.

Video S3 The collective motion of swarming pH-RPNRs in "rolling" mode.

Video S4 The collective motion of swarming pH-RPNRs during prolonged movement and when repeatedly activated and stopped.

Video S5 The collective motion of swarming pH-RPNRs in "walking" mode.

Video S6 The swarming pH-RPNRs passing through a microchannel.

Video S7 The targeted on-the-fly sensing and mapping of local pH by the swarming pH-RPNRs when collectively moving from a pH 7.4 microwell toward an agar gel with pH 4.4.

Video S8 The targeted on-the-fly sensing and mapping of local pH by the swarming pH-RPNRs when collectively moving from a pH 7.4 microwell toward two ager gels with pH 7.4 and pH 4.4, respectively.

Video S9 Targeted on-the-fly pH mapping by swarming pH-RPNRs at the macroscopic level.

Video S10 Targeted on-the-fly temperature sensing and mapping by swarming T-RPNRs when collectively moving toward a heater.

Video S11 Targeted on-the-fly glucose sensing and mapping by the swarming G-RPNRs when collectively moving toward an agar gel with 50 mM glucose.

Video S12 Mapping-guided photothermal treatment toward MCF-7 tumor cells by the swarming pH-RPNRs.
